# Efficacy of Semaglutide in a Subcutaneous and an Oral Formulation

**DOI:** 10.3389/fendo.2021.645617

**Published:** 2021-06-25

**Authors:** Juris J. Meier

**Affiliations:** Diabetes Center Bochum-Hattingen, St. Josef-Hospital, Ruhr-University Bochum, Bochum, Germany

**Keywords:** body weight, glycated hemoglobin (HbA_1c_), efficacy, glucagon-like peptide-1 receptor agonist (GLP-1RA), oral, semaglutide, subcutaneous, type 2 diabetes

## Abstract

Despite the benefits of early and effective glycemic control in the management of type 2 diabetes (T2D), achieving glycated hemoglobin (HbA_1c_) targets is challenging in some patients. Glucagon-like peptide-1 receptor agonists (GLP-1RAs) provide effective reductions in HbA_1c_ and body weight. Semaglutide is the only GLP-1RA that is available in both an injectable and oral formulation. The efficacy of once-weekly subcutaneous semaglutide and once-daily oral semaglutide has been investigated in the global SUSTAIN and PIONEER phase III clinical trial programs in a range of clinical settings, including early T2D managed with diet and exercise only, more established T2D uncontrolled on one to three oral antidiabetic drugs, and advanced disease treated with insulin. Across the SUSTAIN program, once-weekly subcutaneous semaglutide 1.0 mg reduced HbA_1c_ by 1.5–1.8% after 30–56 weeks, which was significantly more than sitagliptin, liraglutide, exenatide extended release, dulaglutide, canagliflozin, or insulin glargine. Across the PIONEER program, once-daily oral semaglutide 14 mg reduced HbA_1c_ by 1.0–1.4%, significantly more than sitagliptin or empagliflozin, and to a similar extent as liraglutide after 26 weeks. In addition, subcutaneous semaglutide reduced body weight significantly more than all active comparators tested, while oral semaglutide reduced body weight more than sitagliptin and liraglutide, and to a similar extent as empagliflozin. Neither formulation of semaglutide has been associated with an increased risk of hypoglycemia and both improve various measures of health-related quality of life. Semaglutide offers the benefits of a highly effective GLP-1RA in both injectable and oral formulations. Selection of the most appropriate formulation can be made on an individual basis to best suit the patient’s preferences and needs.

## Introduction

Evidence from trials and real-world studies in patients with type 2 diabetes (T2D) indicates that the risk of complications may be reduced by providing sustained glycemic control and that near-normal glycated hemoglobin (HbA_1c_) levels should be achieved as early as possible in the T2D trajectory (1, 2). However, achieving and sustaining optimum glycemic control remains challenging in many patients (3), despite treatment advances and the availability of new classes of glucose-lowering agents. In a recent study of 28,315 patients with incident T2D, around half of patients spent the 10 years after diagnosis with HbA_1c_ above desired targets: mean percent time spent with HbA_1c_ ≥7% was 40% in the first 2 years and 61% after 6–10 years (3).

Reasons that may be responsible for the lack of improvement in glucose levels over time include failure to address the complex pathophysiology of T2D, therapeutic inertia leading to delayed treatment intensification, insufficient implementation of lifestyle changes, and poor adherence to and persistence with treatment (4, 5). Most patients should receive metformin initially, but if control is suboptimal after 3–6 months, treatment intensification with another glucose-lowering therapy is required, and selection of subsequent therapies should be made on an individualized basis to meet the specific needs of the patient (4).

Glucagon-like peptide-1 receptor agonists (GLP-1RAs) are a well-established class of glucose-lowering agents that induce glucose-mediated stimulation of insulin secretion, reduce glucagon release, reduce hepatic glucose output, delay gastric emptying, increase satiety, and improve cardiovascular risk factors (6–9). By correcting multiple pathophysiological defects in T2D, GLP-1RAs provide effective glycemic control, with a low risk of hypoglycemia, while reducing body weight, blood pressure, and in some cases, cardiovascular events (6).

Semaglutide is the only GLP-1RA that is available in both an injectable and an oral formulation (10). Once-weekly subcutaneous semaglutide was approved by the US Food and Drug Administration in December 2017 (11) and by the European Medicines Agency in February 2018 (12), while once-daily oral semaglutide was approved in the US in September 2019 (13) and in Europe in April 2020 (14). It was thought that an oral formulation may improve convenience, acceptance, and adherence with GLP-1RA therapy, and may provide an additional option to help increase glycemic target achievement, particularly in patients who are reluctant to initiate injectable medications (10).

This article describes results from global clinical trial programs that established the efficacy of subcutaneous and oral semaglutide in a range of clinical settings and discusses factors that may influence the choice of formulation in individual patients. The safety of subcutaneous and oral semaglutide will be covered in a separate article in this issue (15).

## Design of The Sustain and Pioneer Programs

Both formulations of semaglutide were investigated in comprehensive international clinical development programs. As part of the Semaglutide Unabated Sustainability in Treatment of Type 2 Diabetes (SUSTAIN) program, the efficacy of once-weekly subcutaneous semaglutide was evaluated in over 7,000 patients in six global phase IIIa trials (SUSTAIN 1–6) across the wide spectrum of the T2D disease course (16–21) and in nearly 3,000 patients in four phase IIIb trials (SUSTAIN 7–10) (22–25) (**Table 1**). Oral semaglutide was then investigated in eight global Peptide InnOvatioN for the Early diabEtes tReatment (PIONEER) phase IIIa trials in over 8,000 patients, with similarly broad evaluation in different patient populations who were receiving a range of background medications (26–33) (**Table 1**). Further SUSTAIN and PIONEER trials were conducted in Japanese subjects and are not described in detail here.

**Table 1:** Summary of the designs of the global glycemic efficacy SUSTAIN and PIONEER trials (16–26).

Trial	Treatment arms	Key inclusion criteria	Trial duration; blinding	Primary endpoint
**Trials in early T2D (mean duration 3–4 years)**				
SUSTAIN 1 (*N* = 388)	s.c. semaglutide 0.5 mg OW s.c. semaglutide 1.0 mg OW Placebo OW	Treated with diet and exercise HbA_1c_ 7.0–10.0%	30 weeks; blinded	Change in HbA_1c_ from baseline to week 30
PIONEER 1 (*N* = 703)	Oral semaglutide 3 mg OD Oral semaglutide 7 mg OD Oral semaglutide 14 mg OD Placebo OD	Treated with diet and exercise HbA_1c_ 7.0–9.5%	26 weeks; blinded	Change in HbA_1c_ from baseline to week 26
**Trials in established T2D (mean duration 6–10 years)**				
SUSTAIN 2 (*N* = 1,231)	s.c. semaglutide 0.5 mg OW s.c. semaglutide 1.0 mg OW Sitagliptin 100 mg OD	Treated with met, TZD, or both HbA_1c_ 7.0–10.5%	56 weeks; blinded	Change in HbA_1c_ from baseline to week 56
PIONEER 3 (*N* = 1,864)	Oral semaglutide 3 mg OD Oral semaglutide 7 mg OD Oral semaglutide 14 mg OD Sitagliptin 100 mg OD	Treated with met ± SU HbA_1c_ 7.0–10.5%	78 weeks; blinded	Change in HbA_1c_ from baseline to week 26
PIONEER 7 (*N* = 504)	Oral semaglutide (flexible dose adjustment: 3, 7, or 14 mg) OD Sitagliptin 100 mg OD	Treated with 1–2 from met, TZD, SU, SGLT2i HbA_1c_ 7.5–9.5%	52 weeks; open-label*	Proportion of patients with HbA_1c_ <7.0% at week 52
SUSTAIN 3 (*N* = 813)	s.c. semaglutide 1.0 mg OW Exenatide ER 2.0 mg OW	Treated with 1–2 from met, SU, TZD HbA_1c_ 7.0–10.5%	56 weeks; open-label	Change in HbA_1c_ from baseline to week 56
SUSTAIN 7 (*N* = 1,201)	s.c. semaglutide 0.5 mg OW s.c. semaglutide 1.0 mg OW Dulaglutide 0.75 mg OW Dulaglutide 1.5 mg OW	Treated with met HbA_1c_ 7.0–10.5%	40 weeks; open-label	Change in HbA_1c_ from baseline to week 40
SUSTAIN 10 (*N* = 577)	s.c. semaglutide 1.0 mg OW Liraglutide 1.2 mg OD	Treated with 1–3 from met, SU, SGLT2i HbA_1c_ 7.0–11.0%	30 weeks; open-label	Change in HbA_1c_ from baseline to week 30
PIONEER 4 (*N* = 711)	Oral semaglutide 14 mg OD Liraglutide 1.8 mg OD Placebo OD	Treated with met ± SGLT2i HbA_1c_ 7.0–9.5%	52 weeks; blinded	Change in HbA_1c_ from baseline to week 26
SUSTAIN 9 (*N* = 302)	s.c. semaglutide 1.0 mg OW Placebo OW	Treated with SGLT2i ± (met or SU) HbA_1c_ 7.0–10.0%	30 weeks; blinded	Change in HbA_1c_ from baseline to week 30
SUSTAIN 8 (*N* = 788)	s.c. semaglutide 1.0 mg OW Canagliflozin 300 mg OD	Treated with met HbA_1c_ 7.0–10.5%	52 weeks; blinded	Change in HbA_1c_ from baseline to week 52
PIONEER 2 (*N* = 822)	Oral semaglutide 14 mg OD Empagliflozin 25 mg OD	Treated with met HbA_1c_ 7.0–10.5%	52 weeks; open-label	Change in HbA_1c_ from baseline to week 26
SUSTAIN 4 (*N* = 1,089)	s.c. semaglutide 0.5 mg OW s.c. semaglutide 1.0 mg OW Insulin glargine OD	Treated with met ± SU HbA_1c_ 7.0–10.0%	30 weeks; open-label	Change in HbA_1c_ from baseline to week 30
**Trials in advanced T2D (mean duration 13–15 years)**				
SUSTAIN 5 (*N* = 397)	s.c. semaglutide 0.5 mg OW s.c. semaglutide 1.0 mg OW Placebo OW	Treated with basal insulin ± met HbA_1c_ 7.0–10.0%	30 weeks; blinded	Change in HbA_1c_ from baseline to week 30
PIONEER 8 (*N* = 731)	Oral semaglutide 3 mg OD Oral semaglutide 7 mg OD Oral semaglutide 14 mg OD Placebo OD	Treated with basal, basal-bolus, or premixed insulin ± met HbA_1c_ 7.0–9.5%	52 weeks; blinded	Change in HbA_1c_ from baseline to week 26
PIONEER 5 (*N* = 324)	Oral semaglutide 14 mg OD Placebo OD	Moderate renal impairment Treated with met or SU, or both, or basal insulin ± met HbA_1c_ 7.0–9.5%	26 weeks; blinded	Change in HbA_1c_ from baseline to week 26

*With 52-week extension study.

ER, extended release; HbA_1c_, glycated hemoglobin; met, metformin; N, number of randomized patients; OD, once daily; OW, once weekly; s.c., subcutaneous; SGLT2i, sodium-glucose co-transporter-2 inhibitor; sita, sitagliptin; SU, sulfonylurea; T2D, type 2 diabetes; TZD, thiazolidinedione.

Patients with early T2D (mean diabetes duration 3–4 years) managed on diet and exercise only were studied in SUSTAIN 1 and PIONEER 1 (16, 27). Effects in patients with more established T2D (mean diabetes duration 7–10 years) already receiving one to three oral antidiabetic drugs (OADs) and in need of treatment intensification were studied in seven SUSTAIN trials and four PIONEER trials (17–19, 22–25, 28–30, 33). Patients with advanced disease (mean diabetes duration 13–15 years) on insulin who required additional treatment were studied in SUSTAIN 5 and PIONEER 8 (20, 26). Typical inclusion criteria for the SUSTAIN and PIONEER trials were age ≥18 years, a diagnosis of T2D at least 90 days prior to screening, and inadequate glycemic control within a specified HbA_1c_ range (**Table 1**).

In both trial programs, initial dose escalation of semaglutide was implemented to mitigate gastrointestinal adverse events. The SUSTAIN trials assessed final once-weekly doses of 1.0 mg only, or 0.5 mg and 1.0 mg, of subcutaneous semaglutide (16–25). Once-daily doses of oral semaglutide (14 mg only or 3 mg, 7 mg, and 14 mg) were assessed in most trials in the PIONEER program (26–32); however, the 3 mg dose is not approved as a maintenance dose and data are not included here. PIONEER 7 evaluated a flexible dosing approach by which the oral semaglutide dose was adjusted (3 mg, 7 mg, or 14 mg) depending on the patient’s glycemic response and gastrointestinal tolerability, to mimic the individualized approach that may be used in clinical practice (33).

Once-weekly subcutaneous semaglutide was compared with placebo (16, 24), as well as commonly used glucose-lowering agents from drug classes recommended for patients who require further treatment intensification: the dipeptidyl peptidase-4 inhibitor sitagliptin (17); other GLP-1RAs (exenatide extended release [ER], dulaglutide and liraglutide) (18, 22, 25); the sodium-glucose co-transporter-2 inhibitor (SGLT2i) canagliflozin (23); and basal insulin (insulin glargine) (19). In the PIONEER program, four trials compared once-daily oral semaglutide with the active comparators sitagliptin, the SGLT2i empagliflozin, and liraglutide (28–30, 33).

Across the SUSTAIN program, the primary and confirmatory secondary endpoints for most trials were change from baseline in HbA_1c_ and body weight, respectively, to the end of treatment (30, 40, 52, or 56 weeks) (16–25). In the PIONEER program, most trials had the primary and confirmatory secondary endpoints at week 26 of change from baseline in HbA_1c_ and body weight, respectively (26–32). An exception was PIONEER 7, in which the primary endpoint was the proportion of patients achieving HbA_1c_ <7.0% at week 52 (33).

The effects of semaglutide were investigated in certain special populations. SUSTAIN 6 and PIONEER 6 assessed the effects of semaglutide vs. placebo on cardiovascular outcomes in patients with T2D at high risk of cardiovascular events (21, 32), and are discussed in a separate article (34). The PIONEER 5 trial was conducted to explore the efficacy and safety of oral semaglutide 14 mg vs. placebo in patients with T2D (most commonly at an advanced stage) and moderate renal impairment (estimated glomerular filtration rate of 30–59 mL/min per 1.73 m²) (31).

In the SUSTAIN program, analyses were performed on data obtained before the initiation of any rescue medication or before premature treatment discontinuation (16–25). The PIONEER program adopted a different approach, with two scientific questions related to the efficacy objectives being addressed through the definition of two estimands (35). The primary estimand was the treatment policy estimand, presented here, which evaluated the treatment effect for all randomized patients regardless of trial product discontinuation or use of rescue medication. The trial product estimand evaluated the treatment effect, assuming that all patients remained on the trial product for the entire planned trial duration and did not use rescue medication.

## HBA_1C_ Reductions With Semaglutide

**Figure 1:**
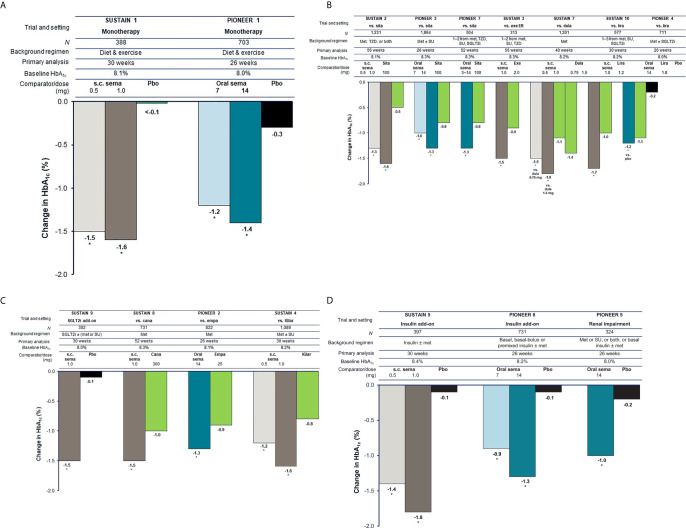
Reduction in HbA_1c_ with semaglutide and comparators in global glycemic efficacy trials (16–20, 22–26, 31, 33). **(A)** Trials in early T2D (mean duration 3–4 years). **(B)** Trials in established T2D (mean duration 6–10 years) with incretin-based therapies as comparators. **(C)** Trials in established T2D (mean duration 6–10 years) with other comparators. **(D)** Trials in advanced T2D (mean duration 13–15 years). For the SUSTAIN trials shown, HbA_1c_ reduction at study end (weeks 30, 40, 52, or 56) was the primary endpoint. Estimated mean changes from baseline in HbA_1c_ included only data obtained before initiation of any rescue medication or before premature treatment discontinuation. For the PIONEER trials shown, HbA_1c_ reduction at week 26 was the primary endpoint, except for PIONEER 7 where the primary endpoint was achievement of HbA_1c_ <7.0% (53 mmol/mol) at week 52. Estimated mean changes from baseline in HbA_1c_ are regardless of trial product discontinuation or rescue medication (treatment policy estimand). Oral semaglutide 3 mg daily was also tested in PIONEER 1, PIONEER 3, and PIONEER 8; however, this dose is not recommended as a maintenance dose [Rybelsus SPC] and data are not shown (except for in PIONEER 7 as part of a flexible dosing approach in which investigators could increase or decrease the dose of oral semaglutide between 3, 7 and 14 mg according to efficacy and tolerability criteria and clinical judgment). *p < 0.05 for the estimated treatment difference with semaglutide vs. comparator. Cana, canagliflozin; dula, dulaglutide; empa, empagliflozin; ER, extended release; exe, exenatide; HbA_1c_, glycated hemoglobin; IGlar, insulin glargine; lira, liraglutide; met, metformin; N, number of randomized patients; pbo, placebo; s.c., subcutaneous; sema, semaglutide; SGLT2i, sodium-glucose co-transporter-2 inhibitor; sita, sitagliptin; SU, sulfonylurea; T2D, type 2 diabetes; TZD, thiazolidinedione.

Results for HbA_1c_ reductions from baseline are shown in **Figure 1**. It should be noted that the SUSTAIN and PIONEER trials differed in their inclusion criteria (e.g., baseline HbA_1c_ and background medication), duration, and analysis approach, therefore the magnitude of HbA_1c_ reduction cannot be directly compared.

### Patients with Early T2D Being Treated With Diet and Exercise

In trials of patients with early T2D insufficiently controlled with diet and exercise alone, who had baseline HbA_1c_ levels of 8.0–8.1%, the highest doses of subcutaneous semaglutide (1.0 mg) or oral semaglutide (14 mg) given as monotherapy were able to reduce HbA_1c_ by 1.6% (at 30 weeks) and 1.4% (at 26 weeks), respectively, and were superior to placebo (both *p* < 0.001) (**Figure 1A**) (16, 27).

### Patients With Established T2D Being Treated With One to Three OADs

Considerable HbA_1c_ reductions (1.0–1.6%) were seen with semaglutide in patients with established T2D who were already receiving one to two OADs in SUSTAIN 2 (metformin ± a thiazolidinedione) and PIONEER 3 (metformin ± a sulfonylurea) (**Figure 1B**) (17, 29). In these trials, subcutaneous semaglutide (0.5 mg and 1.0 mg over 56 weeks) and oral semaglutide (7 mg and 14 mg over 26 weeks) reduced HbA_1c_ significantly more than the active comparator, once-daily sitagliptin 100 mg (all *p* < 0.001) (17, 29). A similar result was observed when flexibly dosed oral semaglutide was compared with sitagliptin over 52 weeks in PIONEER 7 (–1.3 vs. –0.8%; *p* < 0.001) (**Figure 1B**) (33).

When compared with other GLP-1RAs in patients with established T2D already receiving one to three OADs, subcutaneous semaglutide 1.0 mg reduced HbA_1c_ significantly more than once-weekly exenatide ER 2.0 mg (–1.5% vs. –0.9%), once-weekly dulaglutide 1.5 mg (–1.8% vs. –1.4%), and once-daily liraglutide 1.2 mg (–1.7% vs. –1.0%) (all *p* < 0.001) (18, 20, 25) (**Figure 1B**). With oral semaglutide, similar HbA_1c_ reductions were seen as with once-daily liraglutide 1.8 mg when patients were on a background of metformin ± an SGLT2i in PIONEER 4 (–1.2% vs. –1.1%) (30).

When added to an SGLT2i ± metformin or sulfonylurea, subcutaneous semaglutide reduced HbA_1c_ by 1.5% compared with 0.1% with placebo (*p* < 0.001) at 30 weeks in SUSTAIN 9 (**Figure 1C**) (24). When compared with SGLT2i as second-line therapy, subcutaneous semaglutide 1.0 mg reduced HbA_1c_ significantly more than canagliflozin 300 mg after 52 weeks (–1.5% vs. –1.0%; *p* < 0.001), while oral semaglutide 14 mg reduced HbA_1c_ significantly more than empagliflozin 25 mg after 26 weeks (–1.3% vs. –0.9%; *p* < 0.001) (**Figure 1C**) (23, 28). Subcutaneous semaglutide has also been compared with basal insulin. In SUSTAIN 4, in patients uncontrolled on metformin ± a sulfonylurea, subcutaneous semaglutide 0.5 mg and 1.0 mg produced greater HbA_1c_ reductions than insulin glargine over 30 weeks (–1.2% and –1.6% vs. –0.8%; both *p* < 0.0001) (19).

### Patients With Advanced T2D

For patients with advanced uncontrolled T2D already receiving insulin, subcutaneous semaglutide (0.5 mg and 1.0 mg) and oral semaglutide (7 mg and 14 mg) both reduced HbA_1c_ significantly more than placebo (*p* < 0.001) (**Figure 1D**) (20, 26). In SUSTAIN 5, insulin dose decreased from baseline to week 30 with subcutaneous semaglutide 0.5 mg, semaglutide 1.0 mg, and placebo (geometric means from 39.3 to 35.4, from 37.4 to 31.5, and from 36.6 to 35.2 IU, respectively) (20). In PIONEER 8, total daily insulin dose significantly decreased from baseline to week 26 with oral semaglutide 7 mg and 14 mg compared with placebo (–8 IU and –9 IU vs. –1 IU; both *p* < 0.001) (26).

In patients with mean T2D duration of 14 years and with moderate renal impairment in PIONEER 5, oral semaglutide 14 mg was significantly more effective than placebo in reducing HbA_1c_ at 26 weeks (–1.0% vs. –0.2%; *p* < 0.001) (**Figure 1D**) (31).

### Achievement of Glycemic Targets

For both formulations, effective HbA_1c_ reductions allowed the majority of patients to achieve glycemic targets. In the SUSTAIN program, 66–80% achieved HbA_1c_ <7% with subcutaneous semaglutide 1.0 mg, while 55–77% achieved HbA_1c_ <7% with oral semaglutide 14 mg in the PIONEER program (16–20, 22–31, 33).

## Body Weight Reductions With Semaglutide

### Patients With Early T2D Being Treated With Diet and Exercise

**Figure 2:**
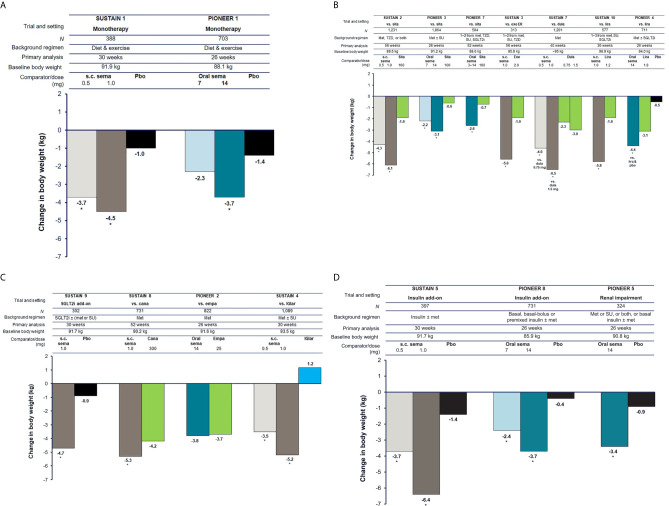
Reduction in body weight with semaglutide and comparators (16–20, 22–26, 31, 33). **(A)** Trials in early T2D (3–4 years). **(B)** Trials in established T2D (6–10 years) with incretin-based therapies as comparators. **(C)** Trials in established T2D (6–10 years) with other comparators. **(D)** Trials in advanced T2D (13–15 years). For the SUSTAIN trials shown, estimated mean changes from baseline in body weight included only data obtained before initiation of any rescue medication or before premature treatment discontinuation. For the PIONEER trials shown, estimated mean changes from baseline in body weight are regardless of trial product discontinuation or rescue medication (treatment policy estimand). Oral semaglutide 3 mg daily was also tested in PIONEER 1, PIONEER 3, and PIONEER 8; however, this dose is not recommended as a maintenance dose [Rybelsus SPC] and data are not shown (except for in PIONEER 7 as part of a flexible dosing approach in which investigators could increase or decrease the dose of oral semaglutide between 3, 7 and 14 mg according to efficacy and tolerability criteria and clinical judgment). *p < 0.05 for the estimated treatment difference with semaglutide vs. comparator. Cana, canagliflozin; dula, dulaglutide; empa, empagliflozin; ER, extended release; exe, exenatide; IGlar, insulin glargine; lira, liraglutide; met, metformin; pbo, placebo; s.c., subcutaneous; sema, semaglutide; SGLT2i, sodium-glucose co-transporter-2 inhibitor; sita, sitagliptin; SU, sulfonylurea; T2D, type 2 diabetes; TZD, thiazolidinedione.

In patients with early T2D, subcutaneous semaglutide 1.0 mg and oral semaglutide 14 mg monotherapy were able to reduce body weight by 4.5 kg and 3.7 kg, respectively, which were superior to the reductions seen with placebo (1.0 and 1.4 kg, respectively) (*p* < 0.001) (**Figure 2A**) (16, 27).

### Patients With Established T2D Being Treated With One to Three OADs

In the SUSTAIN 2, PIONEER 3, and PIONEER 7 trials in patients with established T2D receiving one or two OADs, subcutaneous semaglutide (0.5 mg and 1.0 mg) and oral semaglutide (7 mg, 14 mg, and flexibly dosed) reduced body weight significantly more than sitagliptin (all *p* < 0.001) (**Figure 2B**) (17, 29, 33). When compared with other GLP-1RAs in patients with established T2D, subcutaneous semaglutide 1.0 mg significantly reduced body weight more than once-weekly exenatide ER 2.0 mg (–5.6 kg vs. –1.9 kg), once-weekly dulaglutide 1.5 mg (–6.5 kg vs. –3.0 kg), and once-daily liraglutide 1.2 mg (–5.8 kg vs. –1.9 kg) (all *p* < 0.001) (**Figure 2B**) (18, 22, 25). Oral semaglutide 14 mg reduced body weight significantly more than liraglutide 1.8 mg in PIONEER 4 (–4.4 kg vs. –3.1 kg; *p* < 0.001) (30).

When added to SGLT2i background therapy, subcutaneous semaglutide 1.0 mg reduced body weight by 4.7 kg compared with 0.9 kg with placebo (*p* < 0.001) in SUSTAIN 9 (24) (**Figure 2C**). When compared with SGLT2i as second-line therapy, subcutaneous semaglutide 1.0 mg reduced body weight significantly more than canagliflozin 300 mg at 52 weeks (–5.3 kg vs. –4.2 kg; *p* < 0.01), while oral semaglutide 14 mg produced similar body weight reductions as empagliflozin 25 mg at 26 weeks (–3.8 kg vs. –3.7 kg) (**Figure 2C**) (23, 28). In SUSTAIN 4, patients on one or two OADs who received subcutaneous semaglutide 1.0 mg lost 5.2 kg compared with weight gain of 1.2 kg with insulin glargine after 30 weeks (*p* < 0.001) (19).

### Patients With Advanced T2D

In advanced T2D, both subcutaneous semaglutide (0.5 mg and 1.0 mg) and oral semaglutide (7 mg and 14 mg) reduced body weight significantly more than placebo in patients inadequately controlled on insulin (*p* < 0.001) (**Figure 2D**) (20, 26). In PIONEER 5, patients with moderate renal impairment treated with oral semaglutide 14 mg lost 3.4 kg, while those on placebo lost 0.9 kg at 26 weeks (*p* < 0.001) (**Figure 2D**) (31).

## Patient-Reported Outcomes

Patient-reported outcomes assess psychological aspects such as treatment satisfaction, patient wellbeing, health status, and quality of life to complement clinical outcomes and provide an understanding of the physical, social, and emotional impact of treatment regimens (36).

When treatment satisfaction was measured by the Diabetes Treatment Satisfaction Questionnaire (DTSQ) in patients treated with subcutaneous semaglutide in SUSTAIN 2–5, improvements were significantly greater vs. comparators/placebo (all *p* < 0.05) and were generally greater in patients who achieved vs. did not achieve weight loss and glycemic targets (37). In SUSTAIN 7, improvements in overall treatment satisfaction were generally similar between semaglutide and dulaglutide, irrespective of weight loss or glycemic control.

When the DTSQ was used in PIONEER 4, 7, and 8, total treatment satisfaction scores with oral semaglutide were similar to active comparators and better than with placebo (except in PIONEER 5 in which scores for oral semaglutide and placebo were similar) (26, 30, 31, 33). In PIONEER 4, DTSQ scores favored oral semaglutide over placebo for all items at weeks 26 and 52 except ‘feeling of unacceptably low blood sugars’ (weeks 26 and 52) and ‘flexibility of treatment’ (week 52), which were similar (30). There were no differences in treatment satisfaction between oral semaglutide and liraglutide 1.8 mg.

In PIONEER 7, change from baseline to week 52 in DTSQ scores for satisfaction with treatment, convenience and flexibility of treatment, and total treatment satisfaction appeared similar for oral semaglutide and sitagliptin despite the specific dosing instructions needed with oral semaglutide (33). In PIONEER 5 and 8 in advanced T2D, the frequency of patient-perceived hyperglycemia was significantly lower in the oral semaglutide group than in the placebo group (26, 31).

The 36-item Short-Form Survey (SF-36) version 2 was used to assess physical function, pain, general health, mental health, emotional function, and social function in SUSTAIN 2, 4, and 7 (17, 19, 22). In SUSTAIN 2, several aspects on the SF-36 improved with subcutaneous semaglutide vs. sitagliptin and none worsened (17). In SUSTAIN 4, subcutaneous semaglutide 1.0 mg demonstrated significant improvement compared with insulin glargine in the role-emotional (measure of role limitations due to emotional problems) and general health domains of the SF-36, but not in other domains (19). In SUSTAIN 7, SF-36 scores were similar between subcutaneous semaglutide and dulaglutide (22).

SF-36 version 2 scores were similar between oral semaglutide and sitagliptin in PIONEER 3 and PIONEER 7 (29, 33). In PIONEER 2, scores using the SF-36 were broadly similar with oral semaglutide 14 mg and empagliflozin 25 mg; however, scores were significantly better for oral semaglutide than empagliflozin for the domains of general health and social functioning at week 26, but favored empagliflozin for the role-physical domain and physical component summary scores at week 52 (28). In patients with renal impairment in PIONEER 5, SF-36 scores at week 26 significantly favored oral semaglutide over placebo for the physical component summary and the role-physical, bodily pain, and social functioning domains (31).

For patients with more advanced disease in PIONEER 8, oral semaglutide 14 mg significantly improved general health at week 52 and mental health at week 26 compared with placebo (26). Furthermore, significant improvements in the psychosocial domain and total score of the Impact of Weight on Quality of Life-Lite Clinical Trial Version were observed with oral semaglutide 14 mg vs. placebo at weeks 26 and 52.

## Exposure−Response Relationships

In pharmacokinetic studies, lower bioavailability with oral administration of semaglutide appeared to result in more variable plasma concentrations compared with subcutaneous administration (38, 39). Using data from the SUSTAIN and PIONEER trials, population pharmacokinetic and exposure–response analyses were used to investigate if the oral route of administration changed the efficacy and tolerability of semaglutide compared with subcutaneous administration (39). Exposure−response analyses showed greater HbA_1c_ reductions with increasing semaglutide exposure and the same relationship was observed with body weight reductions. The exposure range with oral semaglutide was found to be wider than for subcutaneous semaglutide, consistent with the more variable plasma concentrations with oral treatment, but there was considerable overlap between oral semaglutide 7 and 14 mg and subcutaneous semaglutide 0.5 and 1.0 mg. The authors concluded that similar exposure−response relationships were observed for efficacy (HbA_1c_ and body weight) and also for tolerability (nausea and vomiting) of semaglutide, regardless of the route of administration.

## Selection of The Most Appropriate Formulation

**Figure 3:**
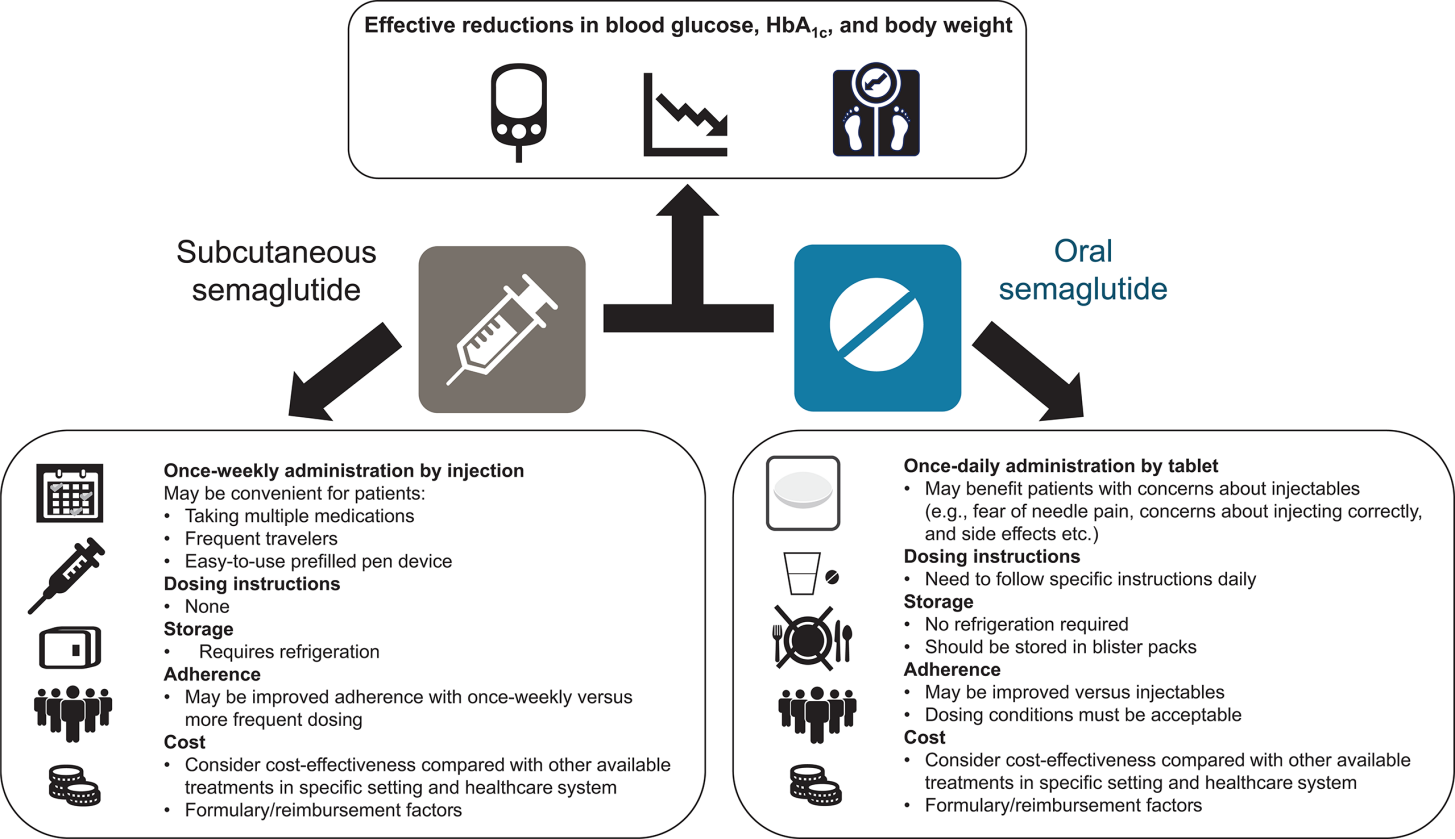
Overview of considerations related to the use of subcutaneous and oral formulations of semaglutide. HbA_1c_, glycated hemoglobin.

With the efficacy of both formulations established and approval granted, healthcare professionals and patients are in a position to choose the formulation that best suits the needs of the individual patient (**Figure 3**).

Regarding efficacy, a network meta-analysis showed that once-daily oral semaglutide 14 mg was associated with numerically greater HbA_1c_ reductions than once-weekly subcutaneous semaglutide 0.5 mg and also dulaglutide 1.5 mg and liraglutide 1.8 mg (40). No statistical difference in efficacy was observed between oral semaglutide 14 mg and once-weekly subcutaneous semaglutide 1.0 mg at week 26, although HbA_1c_ reductions were numerically greater with subcutaneous semaglutide 1.0 mg. Oral semaglutide provided a significantly greater reduction in body weight than all GLP-1RA comparators studied except subcutaneous semaglutide 0.5 mg and 1.0 mg (40). No head-to-head studies have compared approved doses of oral semaglutide (7 mg and 14 mg) vs. once-weekly subcutaneous semaglutide (0.5 mg and 1.0 mg). Doses of oral semaglutide of 2.5 mg, 5 mg, 10 mg, 20 mg, and 40 mg were studied in the phase II trial (41). The phase II trial included an arm in which patients received subcutaneous semaglutide 1.0 mg; however, the primary endpoint of glycemic efficacy was only statistically significant compared with placebo, not between active oral vs. injectable treatment groups (41). In the exposure analyses, average exposure for once-weekly subcutaneous semaglutide 1.0 mg was higher than with oral semaglutide 14 mg, but as mentioned, the exposure range with oral semaglutide was wider than for subcutaneous dosing, with a considerable overlap between oral semaglutide 7 and 14 mg and subcutaneous semaglutide 0.5 and 1.0 mg (39).

As discussed in detail in (15), the risk of hypoglycemia is low with both formulations of semaglutide, despite the effective HbA_1c_ reductions (16–20, 22–31, 33), which may be due to the glucose-dependent mechanism of action of GLP-1RAs. The safety profile is very similar for both formulations (16–20, 22–31, 33). Injection-site reactions are uncommon with the subcutaneous formulation (18). Subcutaneous semaglutide has proven cardiovascular benefit (21); this has not been demonstrated for oral semaglutide, although cardiovascular safety has been shown (32, 34).

Given the generally similar efficacy and safety profiles of the two formulations, other considerations may need to be taken into account when selecting the most appropriate formulation to use. Many patients are reluctant to initiate injectable treatment and barriers to their use include fear of injection pain, feelings of failure related to disease progression, embarrassment/concerns about injecting in public, being nervous about injecting correctly, and adverse events (42, 43). Physicians may also be reluctant to start injectable therapy due to concerns over patient adherence, perceived fear of injection pain, and lack of knowledge of newer therapies (44). For patients who are reluctant to initiate injectable therapy and have a preference for oral administration, oral semaglutide may represent the more appropriate choice. However, the effective use of oral semaglutide depends on the patient following certain dosing instructions. Patients are instructed to swallow the oral semaglutide tablet whole on waking and on an empty stomach, with a sip of water (up to half a glass of water equivalent to 120 mL), and to wait at least 30 minutes before eating, drinking, or taking other oral medications that day (13, 14). The beneficial effects of oral semaglutide may be attenuated if this guidance is not followed.

In a survey for more than 500 patients presented with hypothetical drug profiles, a greater proportion of respondents preferred a once-daily oral treatment with fewer dosing requirements, similar to empagliflozin (41%) or sitagliptin (31%), than a profile corresponding to that of oral semaglutide (11%), citing factors such as fasting and potential gastrointestinal effects (45). However, in an actual clinical trial setting (PIONEER 7), patient-reported satisfaction and treatment convenience were similar between oral semaglutide and sitagliptin (33). Another survey of 600 patients compared preferences regarding once-daily oral semaglutide and a once-weekly injectable GLP-1RA. Three times as many patients preferred the oral to the injectable treatment when initially asked (77% vs 24%), but after they were given more detail on the actual dosing requirements, just over half of respondents indicated a preference for oral semaglutide (46). However, preferences may vary according to factors such as geographical region. For example, a survey of Japanese patients (n=500) found that approximately 90% of patients preferred the profile of once-daily oral semaglutide to that of once-weekly injectable dulaglutide (47).

Some patients may prefer the less frequent once-weekly administration of subcutaneous semaglutide over the need to take a tablet with specific dosing instructions each morning, e.g., those with multiple concomitant medications. Patients generally report a preference for less frequent dosing with injectable GLP-1RAs (48–51), and adherence and persistence rates are improved with once-weekly injectable GLP-1RAs compared with more frequently dosed treatments (52–56). In addition, the subcutaneous version of semaglutide might be preferred for patients prescribed levothyroxine, which should itself be taken in the morning on an empty stomach, half an hour before breakfast (57). The use of an injection pen may also be considered more convenient and less burdensome than the need for daily tablets by some patients, e.g., those who travel frequently. The subcutaneous formulation requires refrigeration, unlike tablets, which may be a factor for some patients.

Cost-effectiveness is also likely to be a consideration. The relative cost-effectiveness of the two semaglutide formulations has not been directly compared. However, both subcutaneous and oral semaglutide have been reported to be more cost-effective and offer lower cost-of-control compared with other injectable GLP-1RAs and oral glucose-lowering drugs, although this may vary between different patient cohorts and healthcare settings (58–64). In addition, switching may be dependent on non-medical decisions outwith the physician’s choice, with a recent expert consensus indicating that non-medical triggers for switching to subcutaneous semaglutide from other GLP-1RAs also included formulary changes and insurance mandates, as well as cost considerations (65).

To conclude, when treatment intensification is needed to improve glycemic control, semaglutide offers the benefits of an effective GLP-1RA in both an injectable and an oral formulation. Selection of the most appropriate formulation can be made on an individual basis to best suit the patient’s preferences and needs.

## Author Contributions

The author was involved with drafting and/or critically reviewing all drafts during the development of the article, and provided final approval for submission.

## Funding

This article was supported by Novo Nordisk, who was provided with the opportunity to perform a medical accuracy review.

## Conflict of Interest

JJM has received lecture honoraria and consulting fees from AstraZeneca, Berlin-Chemie, Boehringer Ingelheim, Eli Lilly, Merck Sharp & Dohme (MSD), Novartis, Novo Nordisk, and Sanofi; has received reimbursement of congress participation fees and travel expenses from MSD, Novo Nordisk, and Sanofi; and has initiated projects supported by Boehringer Ingelheim, MSD, Novo Nordisk, and Sanofi.

The author declares that this article received funding from Novo Nordisk. The funder had the following involvement in the article: medical writing support.

## Abbreviations

Cana, canagliflozin; C_avg_, median semaglutide concentration; DTSQ, Diabetes Treatment Satisfaction Questionnaire; Dula, dulaglutide; Empa, empagliflozin; ER, extended release; Exe, exenatide; GLP-1RA, glucagon-like peptide-1 receptor agonist; HbA_1c_, glycated hemoglobin; IGlar, insulin glargine; Lira, liraglutide; Met, metformin; OAD, oral antidiabetic drug; OD, once daily; OW, once weekly; Pbo, placebo; PIONEER, Peptide InnOvatioN for the Early diabEtes tReatment; s.c., subcutaneous; Sema, semaglutide; SF-36, 36-item Short-Form; SGLT2i, sodium-glucose co-transporter-2 inhibitor; Sita, sitagliptin; SU, sulfonylurea; SUSTAIN, Semaglutide Unabated Sustainability in Treatment of Type 2 Diabetes; T2D, type 2 diabetes; TZD, thiazolidinedione.
